# Prevalence, Virulence and Antimicrobial Susceptibility of *Salmonella* spp., *Yersinia enterocolitica* and *Listeria monocytogenes* in European Wild Boar (*Sus scrofa*) Hunted in Tuscany (Central Italy)

**DOI:** 10.3390/pathogens10020093

**Published:** 2021-01-20

**Authors:** Giovanni Cilia, Barbara Turchi, Filippo Fratini, Stefano Bilei, Teresa Bossù, Maria Laura De Marchis, Domenico Cerri, Maria Irene Pacini, Fabrizio Bertelloni

**Affiliations:** 1 Department of Veterinary Sciences, University of Pisa, 56124 Pisa, Italy; giovanni.cilia@vet.unipi.it (G.C.); barbara.turchi@unipi.it (B.T.); domenico.cerri@unipi.it (D.C.); mariairene.pacini@phd.unipi.it (M.I.P.); fabrizio.bertelloni@unipi.it (F.B.); 2 Istituto Zooprofilattico Sperimentale del Lazio e della Toscana M. Aleandri, 00178 Rome, Italy; stefano.bilei@izslt.it (S.B.); teresa.bossu@izslt.it (T.B.); marialaura.demarchis@izslt.it (M.L.D.M.)

**Keywords:** wild boar, *Salmonella enterica*, *Yersinia enterocolitica*, *Listeria monocytogenes*, antimicrobial resistance, virulence gene

## Abstract

Wild boar is an animal the population of which constantly increases in Europe. This animal plays an important role as a reservoir for several pathogens, including three of the most important zoonoses: salmonellosis, yersiniosis and listeriosis. The aim of this investigation was to evaluate the occurrence of antimicrobial-resistant and virulence factor genes of *Salmonella* spp., *Yersinia enterocolitica* and *Listeria monocytogenes* isolated from wild boar in Tuscany (Central Italy). During two consequent hunting seasons (2018/2019 and 2019/2020), rectal swabs, spleens and livers were collected from 287 hunted wild boar to isolate strains. Each isolate was tested to investigate its antimicrobial resistance and to detect virulence factor genes by PCR. Eighteen *Salmonella* strains (6.27%) were isolated. Of these, 66.7% were resistant to streptomycin, 13.4% to cephalothin, 6.67% to imipenem and one isolate (6.67%) was resistant simultaneously to five antimicrobials. Moreover, the most detected genes were *sopE* (73.4%), *pipB* (66.7%), *sodCI* (53.3%), *spvR* and *spvC* (46.7%). In total, 54 (17.8%) *Yersinia enterocolitica* were isolated; of them, 26 (48.1%), 9 (16.7%), 17 (31.5%), 1 (1.85%) and 1 (1.85%) belonged to biotypes 1, 2, 3, 4 and 5, respectively. All strains (100%) demonstrated resistance to cephalothin and 70.4% to amoxicillin-clavulanic acid, 55.6% to ampicillin, and 37.0% to cefoxitin. Additionally, the most detected genes were *ystA* (25.9%), *inv* (24.1%), *ail* (22.2%), *ystB* (18.5%) and *virF* (14.8%). Finally, only one *Listeria* *monocytogenes* isolate (0.35%) was obtained, belonging to serogroup IVb, serovar 4b, and it was found to be resistant to cefoxitin, cefotaxime and nalidixic acid. The results highlighted the role of wild boar as a carrier for pathogenic and antimicrobial-resistant *Salmonella* spp., *Yersinia enterocolitica* and *Listeria monocytogens*, representing a possible reservoir for domestic animals and human pathogens.

## 1. Introduction

The “One Health” idea started at the beginning of the 2000s. This approach is based on the concept that human and animal health are strictly linked and bound to the health of the ecosystems in which they coexist [1]. This concept is envisaged and implemented by the World Organization for Animal Health (OIE) as a collaborative global approach to understanding the risks for humans and animals, including domestic and wild animals, and ecosystem health as a unique entity [2].

Within the One Health approach, zoonosis plays an important role in the interaction between humans, domestic animals, and wildlife. Following European Food Safety Authority (EFSA) and European Centre for Disease Prevention and Control (ECDC) reports, salmonellosis, yersinosis and listeriosis are three of the most important zoonoses spreading in Europe, with around 91,622, 6823 and 2480 human cases per year, respectively [3]. Moreover, focusing on Italy, the last reported numbers of cases of salmonellosis, yersiniosis and listeriosis in humans were 3635, 14 and 180, respectively [3].

Wild animals could be responsible for this phenomenon due to contact with domestic animals, especially breeds in extensive farms, and to humans, due to their behaviour, which is more anthropized [4,5].

Among the impact of wildlife on the “One Health” approach, wild boar represents an important animal not only because its population constantly increased in number in Europe in the last few decades, but also because this animal is a reservoir for several zoonoses [6,7,8,9,10,11,12,13]. Some human categories, such as hunters, have a high risk of contracting infections from wildlife due to the contact with animal carcasses; however, considering the constant increasing of this animal’s population and its “colonization” of urban or suburban areas, common people could potentially come in contact with them, or with their secretions and excretions [14]. Although the direct transmission from wild boar to humans could be of great importance for public health, the contact and consequent sharing of pathogens between wild and domestic animals could amplify this problems [15]. Indeed, it is well documented that, especially in areas where extensive or semi-extensive farms are present, there is a high possibility of the transmission of bacteria and virus between wild boars and bred animals, in particular domestic pigs, but also ruminants [16,17,18,19,20].

*Salmonella* is a Gram-negative, rod-shaped, flagellated, and facultative type of anaerobes bacterium out of the family Enterobacteriaceae [21,22]. The *Salmonella* genus is divided into two broad species: *Salmonella enterica* and *Salmonella bongori* [21,22]. For *Salmonella enterica*, more than 2600 serovars have been isolated and described, and many of these are causes of illnesses in both humans and animals [21,23,24].

*Yersinia enterocolitica* belong to the Enterobacteriaceae family too, and are the causative agent of yersiniosis, an important zoonosis with symptoms ranging from mild, self-limiting diarrhoea to acute mesenteric lymphadenitis, and can sometimes develop into parenteral forms [25,26,27]. The bacterium has been divided into more than 70 serotypes based on differences in the structure of the somatic antigen, and into six biotypes based on its biochemical characteristics [25,27,28].

*Listeria monocytogenes* is a Gram-positive bacterium, which is facultative intracellular, and it causes listeriosis in humans and animals [29,30,31]. *Listeria monocytogenes* is diffused worldwide and spreads in every environment, such as soil, water and feces [29,30,31]. More than 90% of the *Listeria* infection epidemics and sporadic cases were carried out by strains that belong to 3 (1/2a, 1/2b and 4b) of the 13 serovars [29,32].

Wild boar, as well as domestic pigs, could be a possible reservoir of *Salmonella*, *Yersinia* and *Listeria* [33,34,35,36,37,38,39]. The constant screenings of various zoonotic pathogens are necessary due to the constant increases in wild boar population, and due to the consumption of meat.

The aim of this investigation was to investigate the occurrence of pathogenic and antimicrobial-resistant *Salmonella* spp., *Yersinia enterocolitica* and *Listeria monocytogenes* in the wild boar population in the Tuscany region (Central Italy) during two consecutive hunting seasons.

## 2. Results

**Figure 1:**
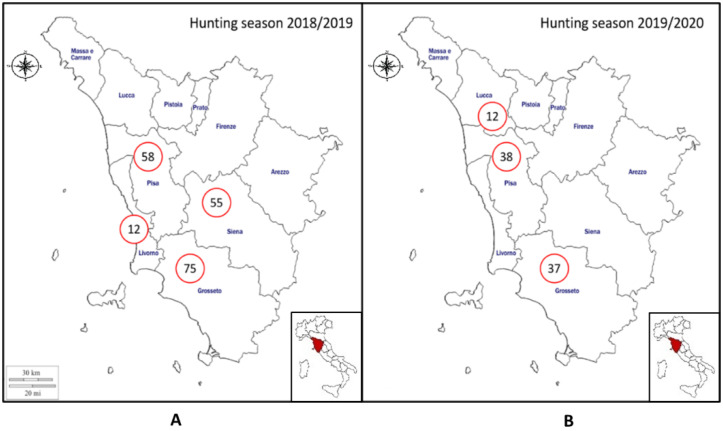
Geographical distribution of the sampling area included in the study (Tuscany region, Central Italy). The number of sampled hunted wild boar per province is indicated in relation to hunting seasons. (**A**) Hunting season 2018/2019. (**B**) Hunting season 2019/2020. (This figure was adapted from Cilia et al., 2020 [40]).

Rectal swabs, spleens and livers were collected from a total of 287 hunted wild boar. In total, 200 wild boar were sampled during the 2018/2019 hunting season (75 from Grosseto province, 58 from Pisa province, 55 from Siena province, and 12 from Livorno province). In addition, 87 animals were sampled during the 2019/2020 hunting seasons (38 from Pisa, 37 from Grosseto and 12 from Lucca) (Figure 1). Sampling was performed in collaboration with hunters, in relation to their availability. For this reason, the sample size could not be predicted beforehand, and for the Siena, Livorno and Lucca provinces, sampling was performed only during one hunting season.

### 2.1. Salmonella *spp.*

#### 2.1.1. Isolation and Characterization

In total, 12 of the 287 (4.18%) animals scored positive, from which 18 Salmonella strains (6.27%) were isolated from collected wild boar samples, and 8 and 10 came from the 2018/2019 and from 2019/2020 hunting seasons, respectively. Detailed serotype characterization and the relationship between each isolate and wild boar organs are reported in Table 1. The isolates included the following: seven Salmonella enterica subspecies diarizonae serotype 50:r:1,5,7; four Salmonella enterica subspecies houtenae serotype 1,40:z4,z23; two Salmonella enterica subspecies enterica serotype Newport; two Salmonella enterica subspecies enterica serotype Kottbus; one Salmonella enterica subspecies enterica serotype London; one Salmonella enterica subspecies enterica serotype Infantis; and one Salmonella enterica subspecies enterica serotype Rubislaw.

#### 2.1.2. Antimicrobial Resistance

Several patterns of antimicrobial resistance have been found in the 18 tested Salmonella strains (Table 1). In total, 10 out of 18 (55.6%) were resistant to streptomycin, 2 of 18 (11.1%) to cephalothin, and 1 of 18 (5.56%) to imipenem. Moreover, one isolate (6.67%) showed resistance simultaneously to tetracycline, enrofloxacin, nitrofurantoin and nalidixic acid, as well as to streptomycin. In total, 5 out of 18 (27.8%) isolates were susceptible to all tested antimicrobials.

#### 2.1.3. Virulence Genes

Excluding the strains S345 and S346, all isolates presented at least one virulence gene (Table 1). The most detected genes were sopE in 11 isolates out of 18 (61.1%), pipB in 10 of 18 (55.6%), and sodCI in 8 of 18 (44.4%). The genes spvR and spvC were found in association in 7 of the 18 isolates (38.9%). A low percentage of Salmonella cultures were positive for sopB (27.8%) and for mgtC (11.1%). None of the tested isolates carried the gene gipA.

### 2.2. Yersinia enterocolitica 

#### 2.2.1. Isolation and Characterization

In total, 71 Yersinia isolates (24.7%) were obtained from wild boar rectal swabs. Only 54 of these latter (18.8% of total) were biochemically confirmed as Yersinia enterocolitica (Table 2), while the other 17 were Yersinia frederiksenii or Yersinia intermedia. In total, 33 and 21 Yersinia enterocolotica cultures were collected during the 2018/2019 and 2019/2020 hunting seasons, respectively. Among the Yersinia enterocolitca isolates, 26 (48.1%), 9 (16.7%), 17 (31.5%), 1 (1.85%) and 1 (1.85%) belonged to biotypes 1, 2, 3, 4 and 5, respectively (Table 2).

#### 2.2.2. Antimicrobial Resistance

High levels of antimicrobial resistance were detected in Yersinia enterocolitica (Table 2). All strains (100%) exhibited resistance to cephalothin. Moreover, resistance was reported in 38 out of 54 (70.4%) for amoxicillin-clavulanic acid, 30 of 54 (55.6%) for ampicillin, and 20 of 54 (37.0%) for cefoxitin. In total, 3 out of 54 (3.70%) isolates were resistant to aztreonam, nalidixic acid and nitrofurantoin. Finally, only 1 of the 54 (1.85%) strains was resistant to streptomycin, cefotaxime, tetracycline and enrofloxacin. None of the isolates showed resistance to imipenem, chloramphenicol, gentamycin, and sulfamethoxazole-trimethoprim.

#### 2.2.3. Virulence Genes

A total of 63.0% of the isolates presented at least one virulence gene (Table 2). The most detected genes were ystA in 14 out of 54 isolates (25.9%), inv in 13 of 54 (24.1%), ail in 12 of 54 (22.2%), ystB in 10 of 54 (18.5%) and virF in 8 of 54 (14.8%).

### 2.3. Listeria monocytogenes

#### 2.3.1. Isolation and Characterization

Only one Listeria monocytogenes isolate (0.35%) was obtained from the 287 collected wild boar. This strain was isolated from the rectal swab of the wild boar C147 during the 2018/2019 hunting season. This isolate belonged to serogroup IVb serovar 4b, as determined by its positive score for genes prfA, ORF2819 and ORF2110, used for molecular characterization.

#### 2.3.2. Antimicrobial Resistance

The only *Listeria monocytogenes* strain showed resistance to cefoxitin, cefotaxime and nalidixic acid.

### 2.4. Statistical Analysis

Concerning the three different pathogens, no statistical differences (*p* > 0.05) were recorded regarding their prevalence in relation to sex, age, hunting seasons and province of sampling.

## 3. Discussion

This investigation confirmed the role of wild boar as a carrier of *Salmonella* spp. and *Yersinia enterocolitica* in the Tuscany region. While, regarding *Listeria monocytogenes*, only one animal was positive, the role of the wild boar in the epidemiology of this bacterium in this area remains unknown, and it probably cannot be considered as a reservoir. Interestingly, only one case of co-infection was recorded (wild boar C263), whereby, from a rectal swab, *Salmonella enterica* subspecies *houtenae* serotype 1,40:z4,z23 and *Yersinia enterocolitica* biotype 1 were isolated.

A total of 4.18% of the sampled wild boar scored positive for *Salmonella* spp. infection. Very similar prevalence was reported in two studies carried out in the Lazio region (7.2%) and in North-West Italy (10.8%) [34,41], as well as in two studies performed in Spain (7.70%) and Sweden (10.0%) [42,43]. On the other hand, in research performed in Switzerland (12.0%) and Portugal (22.0%), the prevalence was higher than that reported in this investigation [44,45,46,47]. Concerning serotype, the most detected strains were *Salmonella enterica* subspecies *diarizonae* serotype 50:r:1,5,7 (7 isolates), followed by subspecies *houtenae* serotype 1,40:z4,z23 (4 isolates), Newport and Kottbus (2 isolates for each serotype) and London, Infantis and Rubislaw (1 isolates for each). *Salmonella* ser. 50:r:1,5,7 and *Salmonella enterica* subspecies *houtenae* serotype 1,40:z4,z23 were also isolated from the wild boar sampled in other northern (Lombardy, Piedmont, Liguria and Valle d’Aosta) and central (Latium) Italian regions, as well as in Spain [34,41,42,48], highlighting the circulation of these *Salmonella* serovars in North-Central Italy and in other Mediterranean countries. On the other hand, the serotype Newport was reported only in the Lombardy region and Spain [42,48], while the serotype Kottbus only in the Lombardy and Latium regions [34,48]. Moreover, the serotype Infantis was previously identified in the wild boar hunted in the North of Italy [41,48]. At the best of the authors’ knowledge, this investigation reported the first isolation in Italy from wild boar samples of serotypes Rubislaw and London.

Half of the positive specimens (six animals) presented multi-organ infection. Indeed C12, C141, C196, C218 and C217 scored positive for *Salmonella* infection in two, or all, of the investigated organs, probably with the same strains. Interestingly, in the spleen and liver of wild boar 209, a co-infection by two different serotypes was reported. None of the isolated *Salmonella enterica* strains proved to be resistant to more than one antibiotic, except for serotype Infantis, isolated from wild boar C103 spleen. The highest percentage of resistance was recorded for streptomycin (55.6%), while low resistances were reported to cephalothin (11.1%) and imipenem (5.56%). These data agree with reports focusing on the antimicrobial resistance of wild boar [34,41,49,50] and swine isolates [23,51,52], especially for the *Salmonella enterica* serovars that are less diffused. Concerning pathogenic characteristics, the presence of some virulence genes, located on *Salmonella* pathogenicity island 3 (SPI-3) and SPI-5, prophages and plasmids, was investigated. The most-detected gene was *sopE*—it was found in three *S.* sub. *diarizonae* ser. 50:r:1,5,7, three *S.* sub. *houtenae* ser. 1,40:z4,z23, two *S.* ser. Kottbus and one *S.* ser. Rubislaw examined strains. Another gene that was highly detected was *sodCI*, found in four *S.* sub. *diarizonae* ser. 50:r:1,5,7, two *S.* sub. *houtenae* ser. 1,40:z4,z23, one *S.* ser. Infantis and one *S.* ser. Rubislaw. The genes *sopE* and *sodCI*, both carried by phages, are more often associated with serotypes Enteritidis and Typhimurium, respectively, and they are rarely detected in other serotypes [23,53,54]. The obtained results expand the bacterial hosts spectrum of these phages, and suggest a possible reservoir for bacteriophages harboring virulence genes among salmonellae circulating in wild animals. The genes *spvRC* were detected in seven isolates belonging to *S.* sub. *houtenae* ser. 1,40:z4,z23, and three *S.* sub. *diarizonae* ser. 50:r:1,5,7. Both genes are part of the *Salmonella* Plasmid Virulence (*spv*) that is associated with more virulent serotypes, such as Typhimurium or Enteritidis. The detection of *spv* in unusual serotypes is not uncommon, but suggests a more wide diffusion of this plasmid and the potential high virulence of these serotypes too [55]. In total, 10 and 5 strains harboured the genes *pipB* and *sopB*, respectively, both located on SPI-5. Only two isolates had these two genes in association, suggesting a possible fragmenting acquisition of SPI-5, which it is not as highly conserved as other SPIs, or the partial genetic leak of this SPI. Finally, only two isolates, one *S.* ser. Rubislaw and one *S.* sub. *diarizonae* ser. 50:r:1,5,7, harboured the gene *mgtC* located on SPI-3. Overall, genes located on prophages and plasmids were detected more frequently than genes located on genomic SPI. This could suggest, as might be expected, a large diffusion of these highly mobile elements.

In this investigation, *Yersinia enterocolitica* was isolated from 18.8% of the samples. In other studies carried out in Sweden, Germany and Poland, the culture-positive prevalence was very similar, ranging from 13.2% to 20% [35,56,57]. On the other hand, in research performed in Japan and Poland the prevalence was higher, from 50% to 74% [36,58,59], while in other studies done in Sweden and in northern Italy, it was lower, at around 7% [43,60]. These data suggest a high variability in isolation rates, probably linked to the different geographic areas and time of sampling. This observation stresses the importance of a constant monitoring of the prevalence of infectious agents among wildlife, which could be influenced by many different factors. Furthermore, the diffusion of *Yersinia enterocolitica* biotypes in wild boar found in this investigation highlighted that biotype 1 was the most prevalent, followed by biotype 3, biotype 2, biotype 4 and biotype 5. These data are in accordance with other previously published work, wherein biotype 1 was the most frequently detected in wild boar [36,39,56,58,59]. The detected *Yersinia enterocolitica* was poorly virulent or non-pathogenic; indeed, most isolates harboured only one or none of the investigated virulence genes. The presence of few virulence genes in *Yersinia enterocolitica* isolates was also reported by other authors, highlighting that wild boar is a *reservoir* for non-pathogenic or less virulent strains [36,58,59,61]. Furthermore, this finding could be linked to the distribution of the detected biotypes. Indeed, in biotypes considered more virulent, as well as biotypes 3 and 5, more than one gene associated with virulence was found. Pathogenic bacteria belonging to this species were found in wild boar specimens, as well as non-pathogenic ones, strengthening the idea, suggested by some studies, that this animal could act as an accidental *Yersinia* carrier [43,60]. In this investigation, the *ystA* virulence gene was the most prevalent, as demonstrated for other wild boar isolates [62]. Moreover, several wild boar isolates scored positive for the *inv* [60], *ail* [59,62,63] and *ystB* [58,61] virulence genes, as obtained from samples here investigated. *virF* is a plasmid regulatory gene located on the virulence plasmid designated pYV; the plasmid genes encoded by this plasmid guide the invasion of *Yersinia enterocolitica* and enable bacteria to survive inside the human host, and for this reasons they are considered essential for pathogenesis [64]. All these observations confirm the low virulence of *Y. enterocolitica* strains circulating among wild boar, despite the high isolation rate. However, most investigated strains had the genes for enterotoxins production, which seem to play an important role in *Yersinia*-induced diarrhoea [65]. Antimicrobial resistance tests revealed a high resistance to penicillins and cephalosporins. In detail, all isolates showed resistance to cephalothin (100%), while high resistances were reported to amoxicillin-clavulanic acid (70.4%), ampicillin (55.6%) and cefoxitin (37.0%). The high resistance to cephalothin was well reported in isolates from domestic swine [66], livestock [67,68] and food [69,70,71]. Moreover, the resistance to penicillins and cephalosporins, including amoxicillin-clavulanic acid, ampicillin and cefoxitin, were also documented in wild boar *Yersinia enterocolitica* isolates [36,56]. For this bacterial species, an intrinsic resistance to β-lactams was suggested, and the obtained results seem to confirm these data [72]. Excluding this class of antimicrobials, a very low level of resistance was recorded among isolates, suggesting the scant involvement of *Yersinia enterocolitica* in this threatening phenomenon.

Only one *Listeria monocytogenes* isolate was obtained from the wild boar rectal swab. The same low infection ratio was previously reported in studies carried out in Japan and the Russian Federation [73,74]. On the other hand, some studies reported a high infection rate, although in these cases isolations from tonsils in particular were recorded [75,76,77,78,79]. These differences could be related to the geographic area, time and sampled organs. Moreover, most of the *Listeria monocytogenes* strains isolated from wild boar belong to serogroup IVb serovar 4b [74,79], as well as the one isolated in this investigation. Usually, the presence of *Listeria monocytogenes* serogroup IVb could be linked to the invasive strains that colonize wild animals that inhabit pristine environments, and so is related to wildlife with little contact with domestic animals and/or humans [79].

## 4. Materials and Methods

### 4.1. Study Area and Sampling

The investigated area (Tuscany region) is a very extensive area which comprises different ecosystems, from mountains to hilly areas, and also reaches the sea. The area, very rich in vegetation, is characterized by sandy coasts, swamps, wetlands, forests, and agriculture and farm areas. Many types of different wild animals are present, in particular wild boars (*Sus scrofa*), foxes (*Vulpes vulpes*), roe deer (*Capreolus capreolus*), fallow deer (*Dama dama*), hares (*Lepus europaeus*), hedgehogs (*Erinaceus europaeus*), badgers (*Meles meles*), porcupines (*Hystrix cristata*), wolf (*Canis lupus*), marmot (*Marmota marmota*), red squirrel (*Sciurus vulgaris*), common rabbit (*Oryctolagus cuniculus*) and different small rodent species, as well as a wide range of birds. Some zones of these areas host farm animals, in particular pig, cattle, sheep and horses, which are mainly bred in extensive or semi-extensive conditions. This area is also characterized by the significant presence of hunting activity, in particular for wild boar. During hunting season, the numbers of this animal specimen that are hunted per year range from 100,000 to 150,000 [80].

During two hunting seasons (from November 2018 to January 2019 and from November 2019 to January 2020), rectal swab, spleen, and liver were collected from wild boar. All specimens were sampled from the Tuscany region (Central Italy), in detail from the provinces of Pisa, Livorno, Siena, and Grosseto. All animals included in the study were hunted during the authorized hunting season, following regional hunting law (Regolamento di Attuazione della Legge Regionale 12 Gennaio 1994, N. 3 D.P.G.R. 48/R/2017). No animals were specifically sacrificed for this study’s purpose. Sampling was performed just before slaughtering procedures, and within 4 h after collection, swabs and organs were transported to the infectious disease laboratory of the Department of Veterinary Sciences, University of Pisa. During sampling, the hunting area, sex, and age of each animal were recorded. In particular, age was evaluated after assessing the degree of tooth eruption and wear of teeth of the lower jaw [81]. Due to the sampling being performed in collaboration with hunter companies, the sample size could not be predicted beforehand, and the authors collected samples from all possible hunted specimens.

### 4.2. Bacterial Isolation and Characterization

*Salmonella* spp. isolation was performed as previously described [23] from collected rectal swabs, spleens and livers. All isolates were serotyped by the “Istituto Zooprofilattico Sperimentale Lazio e Toscana, Rome section”.

*Yersinia enterocolitica* isolation was performed as was earlier reported [82,83] from collected rectal swabs. Biochemical tests were done to distinguish the biotypes of the isolates [25].

*Listeria monocytogenes* isolation was carried out according to Demaître et al. [84] from collected rectal swabs. Suspected *L. monocytogenes* isolates were confirmed by PCR based on the *prfA* gene [85]. Confirmed *L. monocytogenes* isolates were serotyped by multi-step PCR assays, to identify the following serotype groups: IIa (serovars 1/2a and 3a), IIc (serovars 1/2c and 3c), IIb (serovars 1/2b, 3b, and 7), and IVb (serovars 4b, 4d, and 4e) [86,87].

### 4.3. Antimicrobial Resistance

For all *Salmonella* spp., *Yersinia enterocolitica* and *Listeria monocytogenes* isolates, the antimicrobial susceptibility was evaluated using the disc diffusion test on Mueller Hinton Agar (Oxoid, Ltd., Basingstoke, UK) [88]. The following antibiotics (Oxoid) were employed: amoxicillin-clavulanic acid (AMC; 30 µg), ampicillin (AMP; 10 µg), aztreonam (ATM; 30 µg), cephalothin (KF; 30 µg), cefotaxime (CTX; 30 µg), cefoxitin (FOX; 30 µg), chloramphenicol (C; 30 µg), enrofloxacin (ENR; 5 µg), gentamycin (CN; 10 µg), imipenem (IPM; 10 µg), nalidixic acid (NA; 2 µg), nitrofurantoin (F; 300 µg), streptomycin (S; 10 µg), sulfamethoxazole-trimethoprim (STX; 25 µg), and tetracycline (TE; 30 µg). The zone diameter interpretive criteria suggested by Clinical & Laboratory Standards Institute (CLSI) were used [72].

### 4.4. Virulence Genes

From each isolate, DNA was extracted with Quick-DNA Plus Kits (Zymo Research, Irvine, CA, USA), following the manufacturer’s instructions, from overnight bacterial cultures. 

Concerning the *Salmonella* spp. isolated, the presence of the following genes linked to virulence was evaluated using primers and protocols reported by other authors: *mgtC*, *pipB*, *sopB*, *spvR, spvC*, *gipA*, *sodCI*, *sopE* [89,90,91,92,93].

On the other hand, as regards *Yersinia enterocolitica* isolates, the presence of the following virulence genes was evaluated using primers and protocols previously published: *ail*, *virF*, *ystA*, *ystB* and *inv* [94,95,96].

### 4.5. Statistical Analysis

Data were analysed with a chi-square (X^2^) test. The statistical test was used to evaluate the infection ratio of each pathogen in relationship to sex (male or female), age class (young, sub-adult, or adult), province (Pisa, Lucca, Livorno, Grosseto, or Siena) and hunting season (2018/2019 or 2019/2020). The statistical significance threshold was set at a *p* value ≤ 0.05.

## 5. Conclusions

In conclusion, the results of this investigation highlight that a great variability is present among *Salmonella* and *Yersinia enterocolitica* serotypes circulating in free-ranging wild boar. Some strains were more virulent than others, especially the isolates belonging to *Salmonella enterica* subspecies *diarizonae* serotype 50:r:1,5,7, serotype Rubislaw and subspecies *houtenae* serotype 1,40:z4,z23, and *Yersinia enterocolitica* serotypes 3 and 5. Moreover, the single *Listeria monocytogenes* serogroup IVb serovar 4b isolation seems to be strictly related to wild animal infections. Finally, this investigation confirms that many virulent *Salmonella, Yersinia enterocolitica* and *Listeria monocytogenes* strains circulate among wild boar, which represent a source of pathogenic bacteria for humans, especially for hunters and wildlife stakeholders.

## Figures and Tables

**Table 1:** Virulence genes and antimicrobial resistance profiles of analysed and characterized *Salmonella* spp. strains in relation to samples of isolation.

Isolate	Serotype	Source	Wild Boar	Province	Hunting Season	Virulence Genes Profile	Antimicrobial Resistance Profile
S345	Newport	L	C12	Siena	2018/2019		KF
S346	Newport	Sp	C12	Siena	2018/2019		KF
S347	London	R	C51	Grosseto	2018/2019	*pipB*	
S349	Infantis	Sp	C103	Pisa	2018/2019	*sopB*, *sodCI*	TE, ENR, S, F, NA
S352	50:r:1,5,7	L	C141	Grosseto	2018/2019	*sopB*, *sopE*	S
S353	50:r:1,5,7	Sp	C141	Grosseto	2018/2019	*sopB*, *sopE*	S
S354	50:r:1,5,7	Sp	C196	Grosseto	2018/2019	*pipB*, *spvC*, *spvR*, *sodCI*	
S355	50:r:1,5,7	R	C196	Grosseto	2018/2019	*pipB*, *spvC*, *spvR*, *sodCI*	
S382	50:r:1,5,7	Sp	C203	Grosseto	2019/2020	*spvC*, *spvR*	S
S383	50:r:1,5,7	R	C209	Lucca	2019/2020	*sopB*, *pipB*, *mgtC*, *sopE*, *sodCI*	IPM
S387	Rubislaw	L	C209	Lucca	2019/2020	*sopB*, *pipB*, *mgtC*, *sopE*, *sodCI*	S
S386	50:r:1,5,7	R	C216	Grosseto	2019/2020	*mgtC*, *sodCI*	S
S389	Kottbus	R	C218	Grosseto	2019/2020	*pipB*, *sopE*	
S390	Kottbus	Sp	C218	Grosseto	2019/2020	*pipB*, *sopE*	
S391	1,40:z4,z23	R	C263	Lucca	2019/2020	*spvC*, *spvR*	S
S394	1,40:z4,z23	L	C270	Lucca	2019/2020	*spvC*, *spvR*, *sopE*	S
S392	1,40:z4,z23	R	C271	Lucca	2019/2020	*spvC*, *spvR*, *sopE*, *sodCI*	S
S393	1,40:z4,z23	L	C271	Lucca	2019/2020	*spvC*, *spvR*, *sopE*, *sodCI*	S

L: liver; Sp: spleen; R: rectal swab; KF: cephalothin; TE: tetracycline; ENR: enrofloxacin; S: streptomycin; F: nitrofurantoin; NA: nalidixic acid; IPM: imipenem.

**Table 2:** Virulence genes and antimicrobial resistance profiles of analysed *Yersinia enterocolitica* isolated.

Isolate	Biotype	Wild Boar	Province	Hunting Season	Virulence Genes Profile	Antimicrobial Resistance Profile
YC1	1	C10	Siena	2018/2019		AMP, AMC, KF
YC2	1	C11	Siena	2018/2019	*inv*	KF, FOX
YC4	3	C24	Livorno	2018/2019		AMP, AMC, KF, FOX
YC6	3	C36	Grosseto	2018/2019	*ystA*	AMP, AMC, KF, FOX
YC7	3	C37	Grosseto	2018/2019	*ail*	AMP, KF, FOX
YC11	3	C23	Livorno	2018/2019	*virF*	AMP, KF, FOX
YC12	1	C30	Pisa	2018/2019	*ystA*	AMP, KF
YC13	3	C54	Grosseto	2018/2019	*ystA*, *ystB*, *inv*	AMP, AMC, KF, FOX
YC14	3	C53	Grosseto	2018/2019	*ail*	AMP, KF, FOX
YC15	1	C49	Livorno	2018/2019	*virF*	AMP, KF
YC16	1	C74	Pisa	2018/2019	*ystA*	AMP, KF
YC17	1	C92	Pisa	2018/2019		AMP, KF, FOX
YC18	1	C94	Pisa	2018/2019	*ail*, *ystB*	KF
YC20	3	C56	Grosseto	2018/2019	*virF*, *inv*	KF
YC21	2	C48	Siena	2018/2019	*ystA*	KF
YC27	2	C113	Grosseto	2018/2019		AMP, AMC, KF, FOX
YC29	1	C140	Grosseto	2018/2019		AMP, AMC, KF, FOX
YC30	2	C139	Grosseto	2018/2019	*ystA*	AMP, AMC, KF
YC31	3	C124	Siena	2018/2019	*inv*	AMP, KF
YC32	3	C132	Grosseto	2018/2019	*ail*, *ystA*	KF
YC33	5	C134	Grosseto	2018/2019	*ail*, *ystB*, *inv*	KF
YC34	1	C145	Pisa	2018/2019		AMP, AMC, KF, FOX
YC35	3	C146	Pisa	2018/2019	*ail*, *ystA*, *ystB*	AMP, AMC, KF
YC37	2	C149	Pisa	2018/2019		AMP, KF
YC38	2	C150	Pisa	2018/2019		KF, F
YC39	2	C151	Pisa	2018/2019		AMP, AMC, KF, FOX
YC44	3	C163	Grosseto	2018/2019	*ail*, *ystB*	KF
YC45	2	C172	Pisa	2018/2019		KF
YC46	3	C173	Pisa	2018/2019	*ystA*	AMP, KF, S, ATM, NA
YC47	2	C176	Pisa	2018/2019		KF, CTX, TE, ENR, FOX, ATM, NA
YC48	3	C174	Pisa	2018/2019	*inv*	AMP, ATM, KF, FOX
YC49	2	C193	Grosseto	2018/2019	*virF*	AMP, KF
YC50	3	C197	Grosseto	2018/2019		AMP, KF
YC51	3	C202	Grosseto	2019/2020	*ail*, *inv*	AMP, AMC, KF, FOX
YC52	1	C229	Grosseto	2019/2020	*ystA*	AMC, KF
YC53	1	C230	Grosseto	2019/2020		KF, FOX
YC54	1	C232	Pisa	2019/2020		AMP, AMC, KF
YC55	1	C241	Pisa	2019/2020	*virF*	AMP, AMC, KF, FOX
YC56	3	C240	Pisa	2019/2020	*ail*	KF
YC57	4	C244	Pisa	2019/2020	*ystB*, *inv*	AMP, AMC, KF
YC58	1	C249	Grosseto	2019/2020		AMP, AMC, KF, FOX
YC59	1	C252	Pisa	2019/2020	*inv*	AMP, KF
YC60	1	C261	Pisa	2019/2020	*ystA*	AMP, AMC, KF
YC61	1	C258	Pisa	2019/2020	*ystB*, *inv*	AMP, AMC, KF, FOX
YC62	1	C263	Lucca	2019/2020		AMP, AMC, KF, FOX
YC63	3	C262	Lucca	2019/2020	*ail*, *virF*	AMC, KF
YC64	1	C264	Lucca	2019/2020		AMC, KF, FOX
YC65	1	C265	Pisa	2019/2020	*ystA*, *inv*	AMP, AMC, KF, FOX
YC66	1	C269	Pisa	2019/2020		AMP, AMC, KF, FOX
YC67	1	C266	Pisa	2019/2020	*inv*	AMP, AMC, KF
YC68	1	C272	Lucca	2019/2020	*ystA*	AMP, AMC, KF
YC69	1	C275	Lucca	2019/2020		AMP, AMC, KF
YC70	1	C281	Pisa	2019/2020		AMC, KF
YC71	1	C282	Pisa	2019/2020	*ystB*	AMC, KF

AMP: amoxicillin-clavulanic acid; AMC: ampicillin; KF: cephalothin; FOX; cefoxitin; S: streptomycin; ATM: aztreonam; NA: nalidixic acid; CTX: cefotaxime; TE: tetracycline; ENR: enrofloxacin; F: nitrofurantoin.
